# The relationship between IFN-γ, IL-10, IL-6 cytokines, and severity of the condition with serum zinc and Fe in children infected with *Mycoplasma pneumoniae*

**DOI:** 10.1515/med-2024-0987

**Published:** 2024-09-06

**Authors:** Yi Xu, Xiangyong Fei

**Affiliations:** Pediatric Department, Dongyang People’s Hospital, Dongyang, 322100, China; Pharmacy Department, Huai’an Hongze District People’s Hospital, Huai’an, 223100, China

**Keywords:** *Mycoplasma pneumoniae* infection, inflammatory cytokines, severity of the illness, serum trace elements

## Abstract

**Objective:**

To explore the relationship between cytokines such as interferon γ (IFN-γ), interleukin-10 (IL-10), and interleukin-6 (IL-6), as well as the severity of the condition, and serum zinc (Zn) and Fe levels in children with *Mycoplasma pneumoniae* infection.

**Methods:**

A simple random sampling method was used to select 108 children with *Mycoplasma pneumoniae* infection admitted to the hospital from January to December 2022 as the study subjects. Collect demographic data such as gender, age, and course of disease from all patients, as well as inflammatory cytokines (InCs) such as IFN-γ, IL-10, and IL-6, the severity of the condition, and serum trace element information such as Zn, Fe, calcium (Ca), and potassium (K) from all patients. Spearman correlation analysis was used to examine the relationship between IFN-γ, IL-10, IL-6, severity of illness, and Zn, Fe, Ca, K in children infected with *Mycoplasma pneumoniae*. Additionally, receiver operating characteristic (ROC) curve analysis was used to test the predictive efficacy of Zn, Fe, Ca, and K on the severity of the patient’s condition.

**Results:**

This study included 108 children infected with *Mycoplasma pneumoniae*, of whom 6 had clinical data missing >10% and were all excluded. Finally, 102 complete clinical data were collected, with a data recovery efficiency of 94.44%. The differences in IFN-γ, IL-10, IL-6 levels, severity of the condition, as well as Zn, Fe, Ca, K levels among children of different ages, disease courses, body mass, and body temperature showed *P* < 0. 05. Spearman correlation analysis showed that the levels of IFN-γ, IL-10, IL-6, and severity of the condition in children with *Mycoplasma pneumoniae* infection were negatively correlated with Zn, Fe, Ca, and K (*ρ* = −0.319 to −0.827, *P* < 0.05). The ROC curve analysis results indicate that Zn, Fe, Ca, and K can all be used as indicators to predict the severity of the patient’s condition (AUC = 0.710–0.759, *P* < 0.05).

**Conclusion:**

There is a close relationship between InCs and the severity of the condition in children with *Mycoplasma pneumoniae* and serum trace elements. Therefore, clinical attention should be paid to monitoring the serum trace element levels of children, and reasonable measures should be taken to regulate them to accelerate the progress of disease treatment.

## 1 Introduction

*Mycoplasma pneumoniae* infection (MPnI) is a common clinical respiratory disease caused by *Mycoplasma pneumoniae* (MPn), usually affecting children aged 5–15. Its clinical incidence rate is high, accounting for 20–40% of children’s community-acquired pneumonia, and has a rising trend year by year [1,2,3]. After MPn invades the body, it stimulates the production and release of a large number of inflammatory cytokines (InCs). As the level of InCs in the body increases, the severity of the disease (DS) will also worsen. Previous studies have also confirmed a correlation between InCs and MPnIDS [4,5], making it common for clinical evaluation of patients’ DS based on the level of InCs. Trace elements are inorganic substances with concentrations lower than 0.01% of body mass, playing an important role in maintaining the immune system function of the body. Zinc (Zn), Fe, calcium (Ca), and other key trace elements that maintain the normal operation of the body are closely related to cellular signaling, antioxidant response, and immune cell function regulation [6,7]. Research has found that MPnI is related to the imbalance of trace element levels in the body [8]. However, so far, the study about the relationship between InCs and DS in children with MPnI and trace elements is relatively limited. Therefore, this study investigates the correlation between InCs, DS, and trace elements in children with MPnI. It is expected to provide new biological biomarkers and therapeutic targets for the early diagnosis and treatment of MPnI children through this study; the following is reported.

## 2 Objects and methods

### 2.1 Object

The sample size included in investigative studies should be calculated based on 5–10 times the number of clinical data collected. This study collected a total of 14 data items, including 6 demographic data, 3 InCs indicators, 1 DS indicator, and 4 serum trace element (STE) indicators. In addition, considering the possible sample loss in the study, an additional 10% of the samples were included. After calculation, the sample size included in this study should be within the range of 77–154 cases. Therefore, this study used a simple random sampling method to select 108 MPnI patients admitted to the hospital from January 2022 to December 2022 as the study subjects. Inclusion criteria: (1) Comply with the MPnI-related diagnostic standards in the “Chinese Children’s MPnI Laboratory Diagnostic Norms and Clinical Practice Expert Consensus (2019)” [9]; (2) Age < 14 years old. Exclusion criteria: (1) previous history of secondary respiratory diseases such as bronchial asthma and tracheal malformations; (2) severe dysfunction of organs such as heart, liver, and kidney in combination; (3) merge infection with other viruses or pathogens; and (4) combined with systemic immune dysfunction. Removed criteria: (1) missing clinical data; (2) withdrawal from the study midway; and (3) the patient’s condition worsened. Among the 108 children included in this study, 60 were males and 48 were females. Age ranges from 1 to 12 years, with an average of (6.71 ± 2.14) years. The course of the disease is 3–15 days, with an average of (7.83 ± 2.25) days. Body mass is 10.50–37.60 kg, with an average of (22.96 ± 5.78) kg. The body temperature ranges from 37.4 to 41.2°C, with an average of (38.73 ± 0.98)°C. In accordance with the ethical principles of medical research related to human subjects in the Helsinki Declaration of the World Medical Assembly [10], this study has been approved by the Medical Ethics Committee.

### 2.2 Methods

To collect demographic data such as gender, age, course of disease, body mass, and body temperature of the child; collect data on InCs and DS such as interferon γ (IFN-γ), interleukin-10 (IL-10), interleukin-6 (IL-6), as well as STE information such as Zn, Fe, Ca, and potassium (K) at the time of admission of the child.

#### 2.2.1 Detection methods for InCs

On the morning of the patient’s admission, 3 mL of fasting venous blood was taken, and the levels of IFN-γ, IL-10, and IL-6 were analyzed using enzyme-linked immunosorbent assay. The testing kits were all purchased from Shanghai Enzymes Biotechnology Co., Ltd., and the testing steps strictly followed the kit instructions.

#### 2.2.2 Evaluation method of DS

This study divides DS into two levels: mild and severe, both of which fulfill the diagnostic criteria for MPnI outlined in the Chinese Children’s MPnI Laboratory Diagnostic Standards and Clinical Practice Expert Consensus (2019). If one of the following criteria is met on the basis of mild symptoms, it is considered severe: (1) accompanied by obvious shortness of breath or tachycardia, or accompanied by breathing difficulties, cyanosis, etc.; (2) blood oxygen saturation ≤92%; (3) chest X-ray, CT, and other imaging examinations show that the affected area of the lungs is ≥2/3; and (4) complications such as chest effusion, atelectasis, and lung abscess.

#### 2.2.3 Detection methods for STE

On the morning of the patient’s admission, 40 μL of fingertip blood was collected, and the serum Zn, Fe, Ca, and K were analyzed using an iCE^TM^ 3300 AAS atomic absorption spectrometer (produced by Thermo Fisher Scientific) and matching reagents.

### 2.3 Statistical methods

SPSS 26. 0 was used for data processing and analysis during the experiment. The Kolmogorov–Smirnov test confirms that the econometric data in this study all follow a normal distribution, expressed as mean ± standard deviation \[(\bar{x}\pm s)]\] ; Perform independent *t*-tests between the two data groups; Conduct a single factor ANOVA test between multiple data groups; Count the number and percentage of data use cases [*n* (%)], and perform a *χ* ^2^-test; Pearson correlation analysis was conducted on the relationship between InCs and STE in MPnI children; Spearman correlation analysis was conducted on the relationship between DS and STE in MPnI children; Receiver operating characteristic (ROC) curve for predicting the efficacy of STE in predicting DS in children with MPnI; Use GraphPad Prism 9 to draw correlation heat maps and ROC curves. *P* <0.05 indicates a statistically significant difference.


**Ethical approval:** Approval from the Medical Ethics Committee of Dongyang People’s Hospital, Dongren Medical, Protocol Number: 2023-YX-231, Date: 2023.8.2.

## 3 Results

### 3.1 Comparison of InCs levels in children with different demographic characteristics

**Figure 1:**
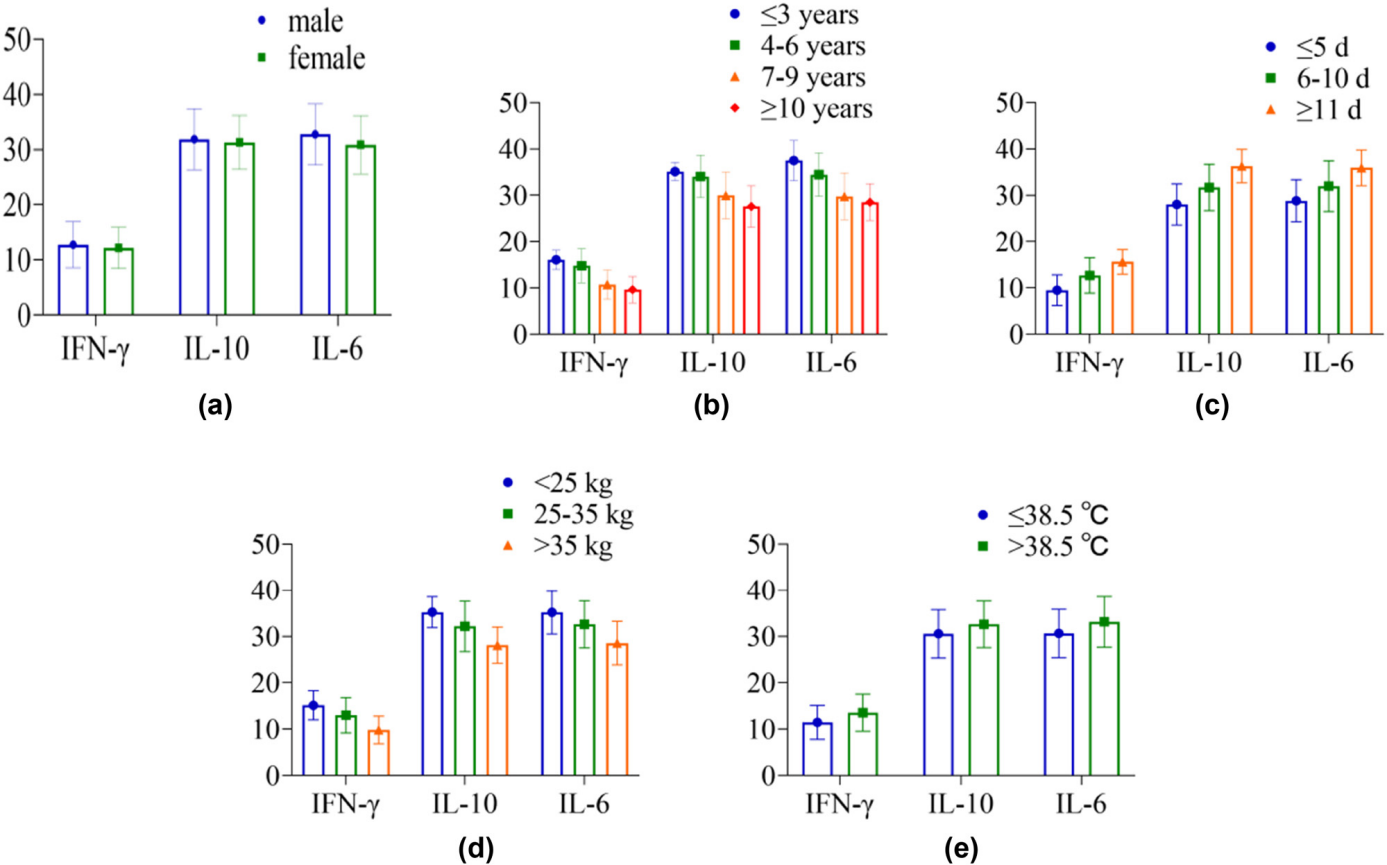
The histogram of InCs in children with different demographic characteristics: (a) gender, (b) age, (c) disease course, (d) body mass, and (e) body temperature.

Among the 108 MPnI patients included, 6 had missing clinical data and were all deleted. Ultimately, 102 complete clinical data were collected, with a data recovery efficiency of 94.44%. In terms of IFN-γ, IL-10, and IL-6 levels in InCs, the difference between children of different genders was *P* > 0.05, and the difference between children of different ages, disease courses, body mass, and body temperature was *P* < 0.05. Table 1 and Figure 1 shows the detailed results.

**Table 1:** Comparative results of InCs in children with different demographic characteristics \[(\bar{x}\pm s)]\]

Demographic characteristics		*n*	IFN-γ (μg/L)	IL-10 (pg/mL)	IL-6 (pg/mL)
Gender	Male	56	12.77 ± 4.15	31.88 ± 5.52	32.76 ± 5.53
	Female	46	12.20 ± 3.72	31.31 ± 4.88	30.89 ± 5.27
*t*			0.728	0.547	1.735
*P*			0.468	0.586	0.086
Age (years)	≤3	6	16.10 ± 2.10	35.14 ± 1.95	37.52 ± 4.33
	4–6	39	14.82 ± 3.66	34.08 ± 4.57	34.49 ± 4.67
	7–9	47	10.75 ± 3.14	29.99 ± 5.03	29.78 ± 5.07
	≥10	10	9.64 ± 2.85	27.60 ± 4.47	28.54 ± 3.96
*F*			15.852	9.058	11.259
*P*			<0.001	<0.001	<0.001
Disease course (days)	≤5	18	9.52 ± 3.30	28.00 ± 4.44	28.82 ± 4.54
	6–10	71	12.70 ± 3.81	31.68 ± 4.99	31.96 ± 5.49
	11≥	13	15.64 ± 2.65	36.30 ± 3.60	35.93 ± 3.86
*F*			11.227	11.541	7.167
*P*			<0.001	<0.001	0.001
Body mass (kg)	<25	28	15.16 ± 3.15	35.24 ± 3.34	35.17 ± 4.65
	25–35	39	13.00 ± 3.80	32.18 ± 5.44	32.60 ± 5.07
	>35	35	9.85 ± 2.98	28.10 ± 3.91	28.55 ± 4.70
*F*			20.041	20.675	15.232
*P*			<0.001	<0.001	<0.001
Body temperature (°C)	≤38.5	53	11.52 ± 3.66	30.63 ± 5.24	30.72 ± 5.23
	>38.5	49	13.59 ± 4.00	32.69 ± 5.04	33.21 ± 5.47
*t*			−2.739	−2.021	−2.355
*P*			0.007	0.046	0.020

### 3.2 Comparison of DS in children with different demographic characteristics

**Figure 2:**
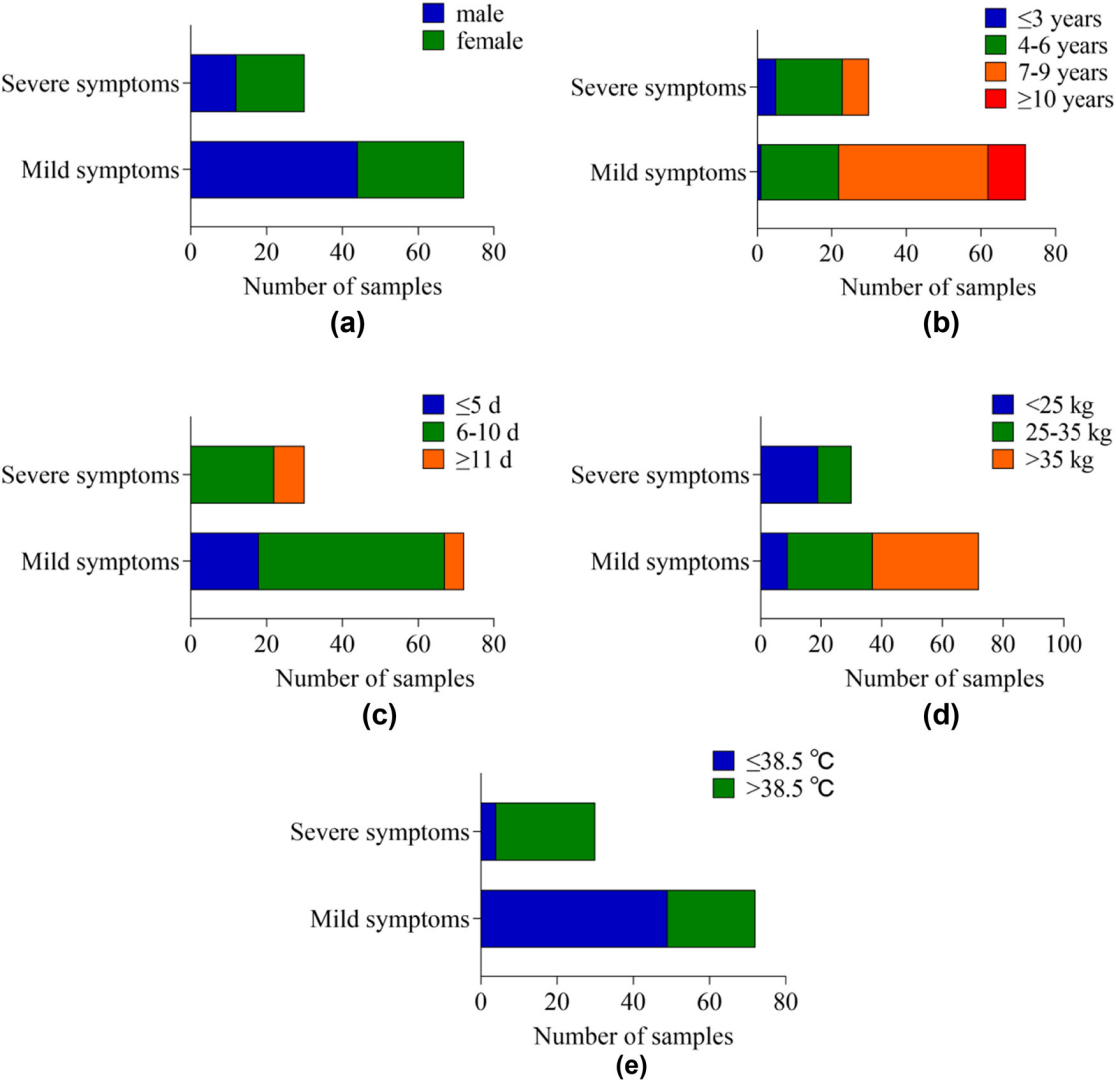
The cumulative bar chart of DS in children with different demographic characteristics: (a) gender, (b) age, (c) disease course, (d) body mass, and (e) body temperature.

In Table 2 and Figure 2, in terms of DS, the difference in DS between children of different genders is *P* > 0.05. The DS of children with different ages, course of disease, body mass, and body temperature was *P* < 0.05.

**Table 2:** Comparison results of DS in children with different demographic characteristics [*n* (%)]

Demographic characteristics		*n*	Mild symptoms	Severe symptoms
Gender	Male	56	44 (78.57)	12 (21.43)
	Female	46	28 (60.87)	18 (39.13)
*X* ^2^			3.812	
*P*			0.051	
Age (years)	≤3	6	1 (16.67)	5 (83.33)
	4–6	39	21 (53.85)	18 (46.15)
	7–9	47	40 (85.11)	7 (14.89)
	≥10	10	10 (100.00)	0 (0.00)
*X* ^2^			22.606	
*P*			<0.001	
Disease course (days)	≤5	18	18 (100.00)	0 (0.00)
	6–10	71	49 (69.01)	22 (30.99)
	≥11	13	5 (38.46)	8 (61.54)
*X* ^2^			14.048	
*P*			<0.001	
Body mass (kg)	<25	28	9 (32.14)	19 (67.86)
	25–35	39	28 (71.79)	11 (28.21)
	>35	35	35 (100.00)	0 (0.00)
*X* ^2^			34.545	
*P*			<0.001	
Body temperature (°C)	≤38.5	53	49 (92.45)	4 (7.55)
	>38.5	49	23 (46.94)	26 (53.06)
*X* ^2^			25.404	
*P*			<0.001	

### 3.3 Comparison of STE levels in children with different demographic characteristics

**Figure 3:**
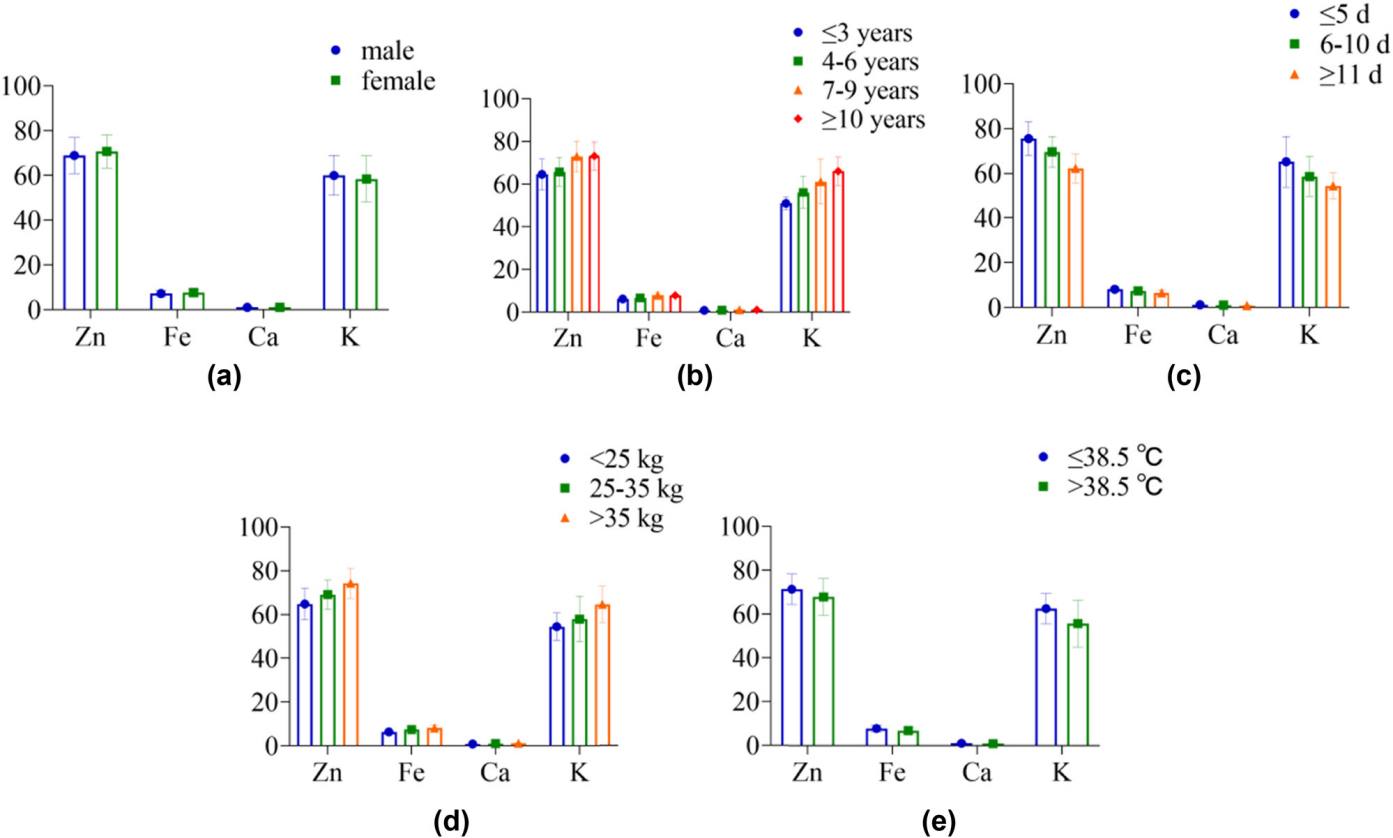
The histogram of STE in children with different demographic characteristics: (a) gender, (b) age, (c) disease course, (d) body mass, and (e) body temperature.

In Table 3 and Figure 3, the levels of Zn, Fe, Ca, and K in STE showed differences between children of different genders (*P* > 0.05) and levels in children of different ages, disease courses, body mass, and body temperature (*P* < 0.05).

**Table 3:** Comparison of STE levels in children with different demographic characteristics \[(\bar{x}\pm s)]\]

Demographic characteristics		*N*	Zn (μmol/L)	Fe (mmol/L)	Ca (mmol/L)	K (mmol/L)
Gender	Male	56	68.89 ± 8.10	7.21 ± 1.44	1.02 ± 0.21	59.99 ± 8.90
	Female	46	70.69 ± 7.36	7.61 ± 1.54	1.04 ± 0.21	58.35 ± 10.35
*T*			−1.160	−1.388	−0.518	0.862
*P*			0.249	0.168	0.605	0.391
Age (years)	≤3	6	64.56 ± 7.17	6.21 ± 0.83	0.89 ± 0.07	51.06 ± 3.05
	4–6	39	65.73 ± 6.69	6.74 ± 1.38	0.93 ± 0.20	56.20 ± 7.48
	7–9	47	72.92 ± 7.23	7.99 ± 1.46	1.11 ± 0.19	61.33 ± 10.65
	≥10	10	73.16 ± 6.56	7.84 ± 0.91	1.14 ± 0.14	66.24 ± 6.58
*F*			9.475	7.954	9.481	6.110
*P*			<0.001	<0.001	<0.001	0.001
Disease course (days)	≤5	18	75.50 ± 7.57	8.16 ± 1.28	1.17 ± 0.15	65.17 ± 11.29
	6–10	71	69.59 ± 6.90	7.35 ± 1.50	1.03 ± 0.21	58.63 ± 9.01
	11≥	13	62.30 ± 6.51	6.57 ± 1.29	0.85 ± 0.13	54.42 ± 5.97
*F*			13.564	4.747	10.716	5.738
*P*			<0.001	0.011	<0.001	0.004
Body mass (kg)	<25	28	64.80 ± 7.10	6.39 ± 1.29	0.89 ± 0.16	54.46 ± 6.25
	25–35	39	69.11 ± 6.76	7.47 ± 1.25	1.01 ± 0.20	57.89 ± 10.45
	>35	35	74.28 ± 6.88	8.11 ± 1.48	1.17 ± 0.15	64.59 ± 8.27
*F*			14.943	12.795	19.494	11.253
*P*			<0.001	<0.001	<0.001	<0.001
Body temperature (°C)	≤38.5	53	71.39 ± 6.90	7.89 ± 1.49	1.10 ± 0.18	62.55 ± 6.99
	>38.5	49	67.87 ± 8.34	6.85 ± 1.30	0.96 ± 0.21	55.67 ± 10.70
*T*			2.329	3.745	3.668	3.745
*P*			0.022	<0.001	<0.001	<0.001

### 3.4 The relationship between InCs, DS, and STE in children with MPnI

**Figure 4:**
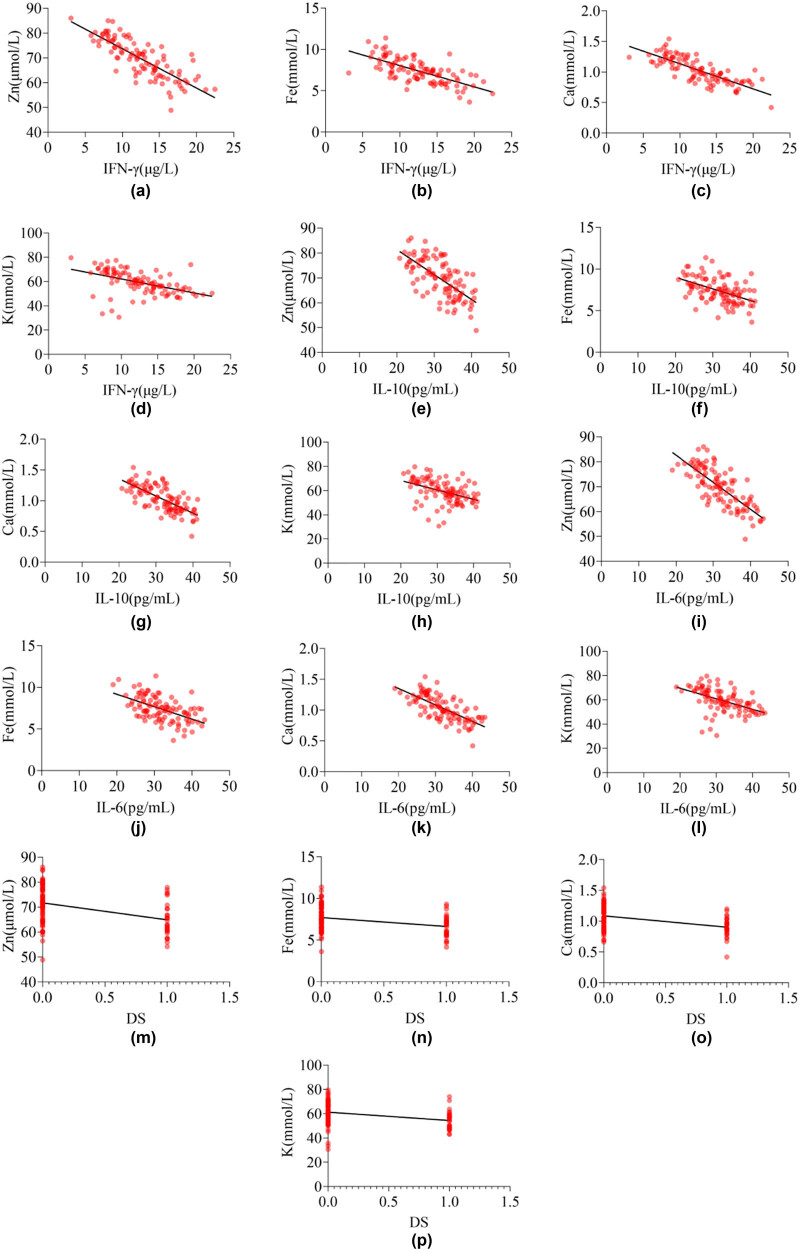
Correlation heatmap of InCs and DS with STE in MPnI children: (a)–(d) the correlation between IFN-γ and STE, (e)–(h) the correlation between IL-10 and STE, (i)–(l) the correlation between IL-6 and STE, and (m)–(p) the correlation between DS and STE.

Due to DS being a non-continuous variable, Spearman correlation analysis was used to test the relationship between DS with STE in children with MPnI, and Pearson correlation analysis was used to test the relationship between InCs with STE in children with MPnI. The results in Table 4 and Figure 4 show that the levels of IFN-γ, IL-10, IL-6, and DS in MPnI children are negatively correlated with Zn, Fe, Ca, and K (*P* < 0.05).

**Table 4:** Relationship between InCs, DS, and STE in MPnI children (*r* and *ρ* value)

		InCs			DS
		IFN-γ	IL-10	IL-6	
Trace element	Zn	−0.827^*^	−0.673^*^	−0.774^*^	−0.395^*^
	Fe	−0.691^*^	−0.501^*^	−0.542^*^	−0.319^*^
	Ca	−0.805^*^	−0.718^*^	−0.736^*^	−0.385^*^
	K	−0.572^*^	−0.474^*^	−0.590^*^	−0.377^*^

Note: *indicates *P* < 0.05.

### 3.5 The predictive efficacy of STE on DS in children with MPnI

**Figure 5:**
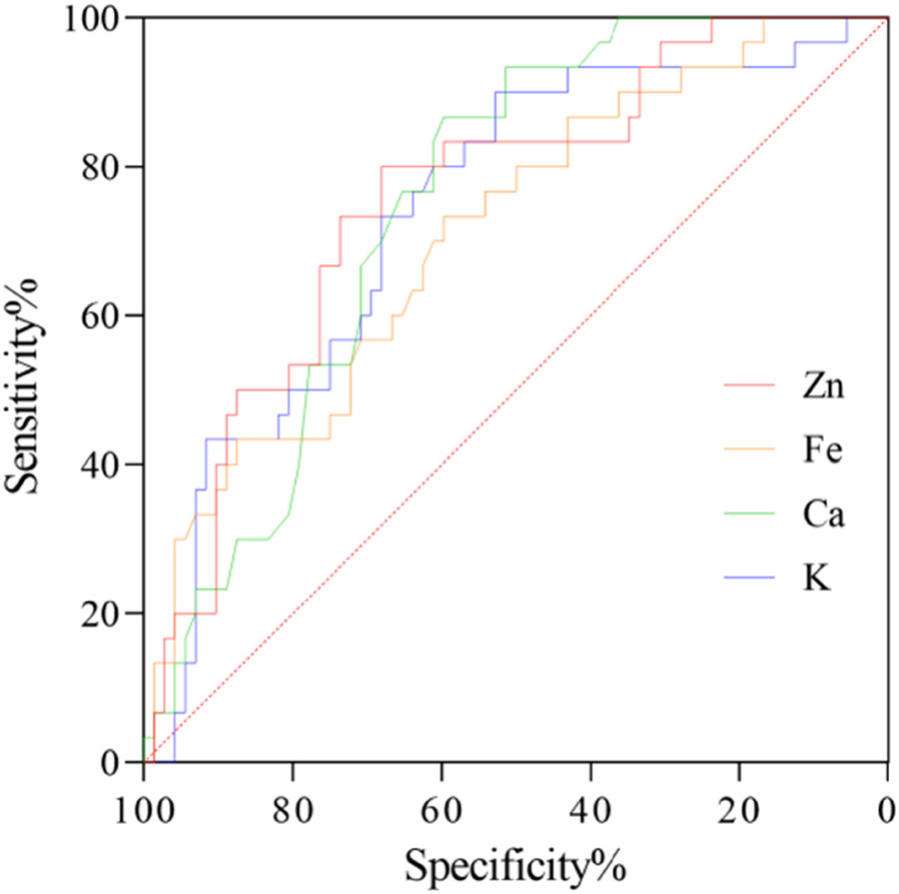
ROC curve of STE on DS in children with MPnI.

To clarify the predictive efficacy of STE on DS in MPn children, this study used DS as a categorical variable and Zn, Fe, Ca, and K as variables to conduct ROC curve analysis. The results of Table 5 and Figure 5 indicate that Zn, Fe, Ca, and K can all be used as indicators for predicting DS in children, with AUC >0.7.

**Table 5:** ROC curve analysis of STE on DS in children with MPnI

Items	AUC	SE	*P*	Youden J	Optimal cutoff value	Sensitivity (%)	Specificity (%)
Zn	0.759	0.052	<0.001	0.481	≤69.36	80.00	68.06
Fe	0.710	0.056	<0.001	0.331	≤7.32	73.33	59.72
Ca	0.751	0.048	<0.001	0.464	≤1.04	86.67	59.72
K	0.741	0.054	<0.001	0.428	≤61.52	90.00	52.78

## 4 Conclusion

Although trace elements have extremely low levels in the human body, they are the core components that make up various enzymes, hormones, vitamins, and other active substances in the human body and are closely related to human health [11].

Zn, as one of the important and essential trace elements in the human body, is involved in the composition of various metal proteins related to DNA replication and cell division. The Zn-rich metalloenzymes and Zn finger proteins in the human body play important roles in promoting growth and tissue regeneration. In addition, Zn also plays a prominent role in maintaining the integrity of the immune system, controlling and preventing infections. When the serum Zn level of children drops, it can lead to the decline of their immune function and an increase of susceptibility, making it easy to induce a variety of infectious diseases [12,13]. In this study, there was a negative correlation between Zn and InCs and DS in children with MPnI (*ρ* = −0.395 to −0.827, *P* < 0.05), and Zn has good predictive power for DS in children (AUC = 0.759, *P* < 0.05). Some studies have pointed out [14] that a decrease in Zn levels in the body can affect protein biosynthesis and the catalytic activity of biological enzymes, while also reducing the body’s immune function. Some scholars have successively used zinc supplementation to treat children with enteritis, and two studies have achieved the same results, that is, the levels of InCs in children are significantly lower than before zinc supplementation [15,16]. Akbari et al. [17] pointed out that MPn can reproduce in the airway epithelium and promote the release of cytokines and inflammatory mediators after invading the body. These factors and neurotransmitters stimulate dendritic cells to induce initial T-cell differentiation toward inhibitory subpopulations. However, Zn deficiency can cause a decrease in T-cell function, leading to impaired humoral immune function and making it difficult to suppress the release of InCs. As the levels of InCs continue to rise, the DS of the child will also worsen. Previous studies have confirmed the relationship between InCs and DS in children with MPnI [18].

Fe is the most abundant essential trace element in the human body. It not only participates in the composition of hemoglobin and myoglobin, as well as the synthesis of related enzymes, but also plays an important role in maintaining normal immune system function and promoting the metabolism of vitamin A and other trace elements. The lack of Fe in the body not only causes anemia, but also affects the immune system, leading to a decrease in the body’s disease resistance. The results of this study show a negative correlation between Fe and the levels of InCs and DS in children with MPnI (*ρ* = −0.319 to −0.691, *P* < 0.05), and Fe has a good predictive effect on DS in children (AUC = 0.710, *P* < 0.05). Research has found that MPnI can cause gastrointestinal dysfunction in children, leading to a decrease in their ability to absorb Fe [19]. This may be one of the reasons why the serum Fe content of MPnI children is lower than that of healthy children. Cho pointed out that compared to mild MPnI, the serum Fe protein levels in children with refractory MPnI are significantly higher [20]. Although the mechanism by which serum Fe protein increases in refractory MPnI is not clear, it may be due to immune response stimulation of monocyte-macrophage proliferation, which enhances its uptake of Fe and hinders its release, leading to an increase in serum Fe protein levels in the body. In addition, the weakened ability of the child to absorb Fe in the gastrointestinal tract further reduces the serum Fe level, which indirectly indicates the relationship between serum Fe level and MPnIDS. In addition, previous studies [21,22] have pointed out that InCs such as IL-10 and IFN-γ can lead to an increase in serum Fe protein levels in the body, which indirectly explains the relationship between serum Fe and InCs.

Ca is one of the essential trace elements in the human body and has important physiological effects. It is an important signaling molecule between cells and regulates immune cell function. The changes in Ca^2+^ concentration in the cytoplasm of immune cells can cause changes in several physiological functions of cells, and the magnitude and duration of the increase in Ca^2+^ concentration in T lymphocytes also determine the intensity and form of the immune response [23]. Therefore, when children lack Ca, it not only leads to symptoms such as convulsions and anorexia, but also affects their immune function. In this study, there was a significant negative correlation between Ca, InCs, and DS in children with MPnI (*ρ* = −0.385 to −0.805, *P* < 0.05), and Ca has good predictive sensitivity and sensitivity to DS (AUC = 0.751, *P* < 0.05). Lv suggests that there may be four possible reasons for the difference in serum Ca levels between mild and severe MPnI children [24]. First, the symptoms of hypoxia in some critically ill children can lead to insufficient energy in the body, leading to the failure of Ca pumps and the opening of Ca channels, leading to Ca influx. Second, defensive protection can cause an increase in nitric oxide levels in the body, and the activity of nitric oxide synthase depends on the activity of Ca-related enzymes, which requires a large amount of Ca influx, leading to a decrease in serum Ca levels in the body. Third, hypoxia in critically ill children can cause a decrease in parathyroid function, a decrease in parathyroid hormone secretion, and a decrease in Ca levels. Fourth, critically ill children may experience disseminated intravascular coagulation, leading to a decrease in free Ca levels in the body. The relationship between serum Ca and InCs may be related to the decrease in immune function caused by Ca deficiency.

K is an important ion that maintains the normal function of cells in the body and is the main cation within cells. It has the function of maintaining physiological functions and regulating immunity and also participates in the activation of various immune cells and cell apoptosis [25]. The research results show that there is a negative correlation between K and InCs and DS in children with MPnI (*ρ* = −0.377 to −0.590, *P* < 0.05), and K has good predictive power for DS in children (AUC = 0.741, *P* < 0.05), which is consistent with the research results in the literature [4]. This study suggests that compared to mild cases, severe cases of MPnI are affected by disease factors leading to insufficient nutrient intake, and gastrointestinal reactions such as diarrhea and vomiting can cause significant loss of K. Inhibition of the respiratory center leads to increased respiratory frequency in children, and excessive lung ventilation leads to respiratory acidosis. At this point, serum K will transfer into the cells, leading to a decrease in serum K levels. As the condition worsens and respiratory depression deepens, serum K levels will also continue to decrease. A decrease in serum K levels will lead to a decrease in the body’s regulatory effect on immune function, weakening the immune system’s control effect on InCs.

In summary, there is a close correlation between InCs and DS in MPn patients and STE, and STE can be used as a new disease evaluation indicator and treatment direction in clinical practice.
